# Deteriorated sleep quality and influencing factors among undergraduates in northern Guizhou, China

**DOI:** 10.7717/peerj.13833

**Published:** 2022-08-24

**Authors:** Yanna Zhou, Shixing Bo, Sujian Ruan, Qingxue Dai, Yingkuan Tian, Xiuquan Shi

**Affiliations:** 1 Department of Epidemiology and Health Statistics, School of Public Health, Zunyi Medical University, Zunyi, Guizhou, China; 2 Comprehensive Ward, Affiliated Hospital of Zunyi Medical University, Zunyi, Guizhou, China; 3 Naxi District Center for Disease Control and Prevention, Luzhou, Sichuan, China

**Keywords:** Sleep quality, College students, Influencing factors

## Abstract

**Background:**

The sleep quality of undergraduates is considerably worse than that in general population, a cross sectional study was conducted to evaluate sleep quality and identify related factors.

**Methods:**

All participants from the freshmen to senior were recruited by the stratified cluster sampling from December 1, 2018 to January 12, 2019. The questionnaire used in this research was primarily composed of three sections: demographic characteristics, Pittsburgh Sleep Quality Index (PSQI) questionnaire and influencing factors of sleep quality. The data were analyzed using SPSS 18.0.

**Results:**

A total of 1,063 valid questionnaires were collected. Among them, 53.7% subjects suffered poor sleep quality. PSQI general score was 5.94 ± 2.73. There were significantly differences in sleep quality in sex, majors and grades. The survey reported that women suffered worse sleep quality than that of men, and medical students suffered worse sleep quality than non-medical students. Meanwhile, it also found that freshmen had better sleep quality than that of sophomores and juniors, sophomores suffered worst sleep quality. The logistic regression analysis showed that bad physical condition (OR (Odds ratio): 2.971 (2.034∼4.339)) and smoking (OR: 1.754 (1.258∼2.446)) were associated with poor sleep quality in males. However, more factors associated with poor sleep quality among females were found, including noisy dormitory environment (OR: 2.025 (1.354-3.030)), skipping breakfast more times per week (OR: 1.332 (1.031∼1.721)), drinking coffee before sleep (OR: 2.111 (1.155∼3.861)), playing with mobile phones for more than 45 minutes before sleep (OR: 1.745 (1.210∼2.515)), more time spent playing games per day (OR: 1.347 (1.048∼1.730)), bad physical condition (OR: 2.507 (1.797-3.497)), and severe academic stress (OR: 1.561 (1.126-2.166)).

**Conclusion:**

About half of college students experienced poor sleep, and poor sleep quality was prevalent in women, medical students, and sophomores. Moreover, there were more risk factors associated with the poor sleep quality of women than with men. Health policymakers should fully consider these factors in improving the sleep quality of college students.

## Introduction

### The definition of sleep disturbances

Sleep disturbances encompassing a wide range of sleep complaints, such as difficulty in initiating sleep (DIS), difficulty in maintaining sleep (DMS), early morning awakenings (EMA), non-restorative sleep (NRS) and poor sleep quality, are a major health problem (Li et al., 2018).

### Prevalence in college students

Previous studies demonstrated that sleep disturbances are common in all around the world, sleep problems might be a unrecognized public health issue in low- and middle-income and emerging economy countries, especially among young adults and university student populations (Peltzer & Pengpid, 2015). For many college students, the transition from adolescence to adulthood poses numerous new challenges, such as roommates/dorm life, erratic living schedules, coping with high academic and social pressures, work obligations, and new level of independence, *etc*. This transition may provide the necessary stress for development or exacerbation of sleep disorder, which is highly prevalent in this population (Jiang et al., 2015; Peltzer & Pengpid, 2015).

### The effect of poor sleep

Sleep disorder is detrimental to physical and mental health of the young generation. The consequences of poor sleep in this population are increasingly salient, as several recent studies have shown poor sleep is associated with poor physical and psychological health outcomes, such as mood disturbances and decreased cognitive performance (Li et al., 2018). Also, poor sleep has been associated with worse academic performance, which can result in dropout or underemployment (Barahona-Correa et al., 2018). Further, there is evidence for a causal association between sleep problems and general overweight or obesity (Fatima, Doi & Al Mamun, 2018; Öztürk & YabancıAyhan, 2017).

### The factors of poor sleep quality

A systematic review reported that 18.5% (95% CI (confidence interval): 11.2∼28.8%) of adolescents had sleep quality problems worldwide, considerably higher than rates of 7.4% reported in general population (Jiang et al., 2015). Research has indicated that sleep quality is linked to smartphone use (Wang et al., 2019). Lifestyle and behaviors, such as eating nutritious foods, may be able to influence sleep quality (St-Onge, Mikic & Pietrolungo, 2016). A study conducted by Sawah et al. (2015) revealed that higher odds of developing poor sleep quality were associated with coffee intake. In a study of 1,044 university students, Kenney et al. (2013) found that students with poor sleep quality had a greater likelihood of alcohol abuse. In addition, the level of sleep quality is also linked to poor physical health and risky health behaviors (Swinkels et al., 2013), excessive Internet use (Shadzi, Salehi & Vardanjani, 2020), and smoking (Cohrs et al., 2014) and physical activity (Lee, 2017). Moreover, many studies showed that cold indoor environments negatively affected sleep comfort and quality (Chimed-Ochir et al., 2021).

To our knowledge, although the foregoing studies verified that poor sleep quality was prevalent among college students and associated with various factors, there was few studies related to sleep quality among undergraduates conducted in the southwest of China, especially in Guizhou province, which is located on the Yunnan-Guizhou Plateau, with a low economic level and relative laggard in many aspects compared to the eastern part of China; moreover, there were significant differences of lifestyle compared with other parts of China. A previous meta-analysis reported that there was one related study conducted in Guiyang published in Chinese in 2011 (Li et al., 2018). Therefore, it is important to evaluate the prevalence of poor sleep quality and explore whether sleep quality is associated with a variety of behaviors and external factors. For this purpose, a cross-sectional study was conducted in a university located at the north of Guizhou Province, China; participants filled out a questionnaire which included evaluation of PSQI scores and related behaviors and external factors. Moreover, previous studies showed there were some difference of life habits between genders, such as smoking (Li et al., 2022; Yu et al., 2022), drinking, personal hygiene, the amount and frequency of exercise (Yang et al., 2022) and the frequency of playing games (Yang et al., 2022), which were all associated with sleep quality; thus, the influence factors between genders should be considered respectively. We hope that the results of this study can lead to a better understanding of sleep quality among college students and what health-promoting activities can improve sleep quality.

## Material & Methods

### Study population and procedure

The cross-sectional study was conducted between December 1, 2018 and January 12, 2019, and the stratified cluster sampling was used to recruit students who were enrolled in Zunyi Medical University, Guizhou province, China, a comprehensive university, including medical major and non-medical major, stratification was carried out according to different grades and majors. Before the survey, the research assistants accepted a unified training, then the research assistants entered the classes, explained the objectives and details of the project and distributed questionnaires, students were given sufficient time to answer the questions independently, and questionnaires were collected on the spot. All participants provided informed written consent and they received monetary compensation for taking part in this study, which was approved by the Medical Ethics Committee of Zunyi Medical University (No.: ZMU-IRB[2018] 1-067). Participants who did not provide informed written consent or did not complete the study questionnaire were excluded from the study. A total of 1,200 questionnaires were distributed, 1,063 valid questionnaires were regained, and the valid rate was 88.6%.

### Questionnaire

A standardized and unified self-report closed-end questionnaire was used for the survey. The questionnaire consisted of three parts, including basic demographic data, the Pittsburgh Sleep Quality Index (PSQI) questionnaire, and the influence factors questionnaire.

The first part included the basic demographic data of age, sex, grade, height, weight and major.

The second part was the PSQI questionnaire which is the most common measure of sleep quality (Pilz et al., 2018). The PSQI is an 18-item self-report questionnaire designed to assess overall sleep quality over last one month period. The first four items relate to sleeping habits and are in free response format (*e.g.*, “How many hours of actual sleep do you get at night?”). The remaining items are related to sleep disturbances and daytime impairments. The response choices include four options referring to the last month (*e.g.*, 0 = Not during the past month, 1 = Less than once a week, 2 = Once or twice a week, 3 = Three or more times a week). The 18 items are scored nonlinearly to generate seven component scores, namely, subjective sleep quality (hereafter referred to as Quality), sleep latency (Latency), sleep duration (Duration), habitual sleep efficiency (Efficiency), sleep disturbances (Disturbances), use of sleeping medication (Medication), and daytime dysfunction (Dysfunction). Each component score has a possible range of zero to three, with higher scores indicating worse sleep. The sum of the component scores yields a PSQI general score of sleep quality which ranges from zero to 21 (Raniti et al., 2018). It is common to classify subjects into two groups: good (PSQI ≤ 5) and poor sleepers (PSQI > 5) (Sawah et al., 2015; Wang et al., 2019; Buysse et al., 1989).

The third part was a self-designed closed-end questionnaire. Previous studies reported that sleep quality was associated with electronic products, physical activity, living conditions, and lifestyle, *etc*. According to the results of communicating with students, almost all college students have electronic products, and some students did not eat breakfast. Additionally, tea and tobacco and alcohol are the three pillar industries of Guizhou province, which can be bought by students easily. Therefore, we hold a point on that the questionnaire should encompass influence factors as follows, such as smoking habits, drinking habits, and coffee intake before sleep (*e.g.*, 1 = Never, 2 = Occasional (Less than twice a month), 3 = Often); night snack intake habits and skipping breakfast habits (*e.g.*, 1 = Never, 2 = One or twice per week, 3 = More than three times per week), physical condition (*e.g.*, 1 = Good, 2 = General, 3 = Bad), monthly expenses of using mobile phones (*e.g.*, 1 = Less than 50 yuan, 2 = More than 50 yuan), how long did you spent on mobile phones before sleep, time spent on playing games (*e.g.*, 1 = Never, 2 = One to two hours per day, 3 = Three to four hours per day, 4 = More than four hours per day), physical exercise (*e.g.*, 1 = More than five times per week, 2 = Three to four times per week, 3 = One or twice per week, 4 = Never), dormitory noise and bright dormitory light (*e.g.*, 1 = No, 2 = Yes), dormitory environmental hygiene (*e.g.*, 1 = Good, 2 = General, 3 = Bad), relationship with classmates and lover (*e.g.*, 1 = Harmony, 2 = General, 3 = Strained), academic stress and employment stress (*e.g.*, 1 = Mild, 2 = Moderate, 3 = Severe) and family economic conditions (*e.g.*, 1 = Affluence, 2 = General, 3 = Poverty).

Additionally, Cronbach’s alpha coefficient was 0.566 for the third part of questionnaire, the KMO coefficient was 0.670, close to 0.70 (*P* < 0.001). The reliability and validity was acceptable.

### Sample size calculation

We assumed that the unknown prevalence rate of sleep disorder was 50 percent, the precision (two-sided) was 0.1, and the confidence level was 0.95, a sample size of 482 could be calculated by PASS 15.0 (NCSS, LLC, Kaysville, Utah, USA). Considering the sample method was cluster sampling, the sample size should be increased by 50 percent, so we needed to recruit at least 723 participants.

### Statistical analyses

Epidata 3.0 (EpiData Association, Odense, Denmark; http://www.epidata.dk/, ) software was used for data entry and error detection, SPSS 18.0 (IBM Corp., Armonk, NY, USA) was used for statistical analyses. All measures variables followed a normal distribution determination. Normal distribution measurement data was described by mean and standard deviation, differences between two groups were tested by two-independent samples *t*-test, one way analysis of variance (one way ANOVA) was used to test differences of multiple groups, followed by LSD or Tamhane multiple comparisons tests. Abnormal distribution measurement data was described by average rank, differences between two groups were tested by Mann–Whitney U test, differences of multiple groups were tested by Kruskal-Wallis test, followed by adjusted test level through Bonferroni multiple comparisons tests. Count data was described by rate. Logistic regression analysis was conducted to determine the impact of variables on sleep quality, and the influence factors were hierarchically analyzed by gender. *α* = 0.05 was taken as the level of significance.

## Results

### Demographic statistics

A total of 1,063 valid questionnaires were collected. Participants were between 17 and 26 years old (mean 19.8 ± 1.3 years): 646 were females and 417 males, 738 were medical students and 325 were non-medical, numbers of freshmen, sophomore, junior and senior were 344, 458, 240, 21, respectively.

#### The analysis of sleep quality among different types of students

High prevalence of poor sleep quality was reported, and the rate of poor sleep quality was 53.7% (571/1063). The average of PSQI general score was 5.94 ± 2.73. We then analyzed the subjects according to sleep quality.

In terms of sex, the results of the *t*-test showed differences of PSQI general score, bedtime, and get up time (*P* < 0.05), the results of the Mann–Whitney U test showed differences in PSQI components, including subjective sleep quality, sleep latency, sleep disturbance, and daytime dysfunction (*P* < 0.05). Sleep quality among females was worse than in males. There were no statistically significant differences found in other variables (Table 1).

**Table 1:** The differences of PSQI general scores, PSQI component scores, and PSQI time variables between genders and majors.

Indices	Gender			Majors		
	Male (*n* = 646)	Female (*n* = 417)	*P* value	Non-medical (*n* = 325)	Medical (*n* = 738)	*P* value
PSQI general score^a^	5.55 (2.83)	6.19 (2.63)	<0.001	5.76 (2.83)	6.02 (2.69)	0.149
PSQI components						
Quality^b^	501.61	551.62	0.004	532.86	531.62	0.946
Latency^b^	496.31	555.04	0.001	505.42	543.71	0.049
Disturbance^b^	498.22	553.80	<0.001	526.65	534.36	0.621
Dysfunction^b^	479.49	565.89	<0.001	492.02	549.61	0.003
Time variables						
Bedtime (hh:mm)^a^	23:53 (00:53)	23:46 (00:47)	0.019	23:55 (00:52)	23:47 (00:48)	0.012
Get up (hh:mm)^a^	07:48 (01:07)	07:41 (00:46)	0.048	07:45 (01:08)	07:43 (00:48)	0.761
Latency (min)^b^	514.89	543.04	0.136	491.98	549.62	0.004

**Notes.**

a All data were reported as mean (standard deviation) using two independent samples *t*-test.

b All data were reported as $\overline{R}$ using the Mann–Whitney *U* test.

In terms of majors, the results of PSQI components showed that the average rank of the sleep latency and daytime dysfunction of medical students was higher than that of non-medical, there were statistically significant differences (*P* < 0.05); In PSQI time variables, bedtime of medical majors was earlier than that of non-medical majors (*P* < 0.05), and latency was longer than that of non-medical majors, they were statistically significant differences. There were no significant differences found in other variables (Table 1).

When compared among grades, the results of one-way ANOVA showed differences of PSQI general score among grades (*P* < 0.05), and both the score of sophomore and junior were higher than that of freshman. In PSQI components, subjective sleep quality, sleep latency, and sleep efficiency showed statistically significant differences among grades (*P* < 0.05), both the average rank of subjective sleep quality and sleep latency of sophomore and junior was higher than those of freshman, the average rank of sleep efficiency of sophomore, junior, and senior was higher than that of freshman, the same to sophomore *vs* junior. In PSQI time variables, there were statistically significant differences in bedtime, wake-up time and latency among grades (*P* < 0.05), the bedtime of sophomore and junior was earlier than that of freshman, sophomores woke up later than freshman, latency of sophomore and junior was longer than that of freshman, the same to sophomore *vs* senior (Table 2).

### The analysis of influence factors related to sleep quality

The results of univariate logistic regression analysis of influence factors for male showed that there were statistically significant differences in dormitory noise, dormitory environmental hygiene, monthly expenses of using mobile phones, time spent on mobile phones before sleep, smoking, coffee intake before sleep, night snack intake, physical condition and frequency of participating in physical exercise (Table 3). Factors with statistically significant differences in univariate analysis were taken as independent variables and sleep quality (PSQI ≤5 = 1, PSQI >5 = 2) as dependent variable. The results of multivariate logistic regression analysis showed that higher odds of developing poor sleep quality were associated with physical condition (OR: 2.971 (2.034∼4.339)) and smoking (OR: 1.754 (1.258∼2.446)) (Table 4).

**Table 2:** The differences of PSQI general scores, PSQI component scores, and PSQI time variables among grades.

	Freshman (*n* = 344)	Sophomore (*n* = 458)	Junior (*n* = 240)	Senior (*n* = 21)	*P* value
**PSQI general score** ^a^	5.47 (2.47)	6.27 (2.91)^*^	6.01 (2.64)^*^	5.62 (2.56)	<0.001
**PSQI components**					
Quality^b^	489.16	550.17^*^	559.24^*^	526.26	0.005
Latency^b^	448.87	577.12^*^	567.99^*^	498.29	<0.001
Efficiency^b^	475.09	572.71^*^	533.02^*^	564.74^*^	<0.001
**Time variables**					
Bedtime (hh:mm)^a^	23:59 (00:46)	23:47 (00:52)^*^	23:41 (00:46)^*^	23:59 (00:53)	<0.001
Get up (hh:mm)^a^	07:36 (00:49)	07:52 (00:51)^*^	07:38 (01:08)	07:52 (00:41)	0.001
Latency (min)^a^	457.11	571.54^*^	564.98^*^	519.57	<0.001

**Notes.**

a All data were reported as mean (standard deviation) using one way ANOVA, *vs Freshman *P* < 0.05.

b All data were reported as $\overline{R}$ using Kruskal-Wallis test, *α*′ = 0.05/6 ≈ 0.0083, *vs Freshman *P* < 0.0083.

The results of univariate logistic regression analysis of influence factors for females indicated statistically significant differences in dormitory noise, bright dormitory light, dormitory environmental hygiene, time spent on mobile phones, time spent on playing games per day, drinking, smoking, coffee intake before sleep, skipping breakfast, physical condition, frequency of participating in physical exercise, relationship with classmates, academic stress, and family economic conditions (Table 3). Factors with statistically significant differences in univariate analysis were taken as independent variables and sleep quality (PSQI ≤5 = 1, PSQI >5 = 2) as dependent variable. The results of multivariate logistic regression analysis showed that the significant influence factors consisted of dormitory noise, skipping breakfast, drinking coffee before sleeping, time spent on mobile phones before sleep, time spent on playing games per day, physical condition and academic stress. Noisy dormitory environment (OR: 2.025 (1.354−3.030)), skipping breakfast more times per week (OR: 1.332 (1.031∼1.721)), drinking coffee before going to bed (OR: 2.111 (1.155∼3.861)), playing mobile phones more than 45 min before going to bed (OR = 1.745 (1.210∼2.515)), more time spent on playing games per day (OR: 1.347 (1.048∼1.730)), bad physical condition (OR: 2.507 (1.797−3.497)) and severe academic stress (OR: 1.561 (1.126−2.166)) were not conducive to ensuring sleep quality (Table 4).

**Table 3:** The univariate logistic regression analysis (OR, 95% CI).

Category	Male	Female
Dormitory noise (1:no, 2:yes)	1.598 (1.076∼2.375)	1.979 (1.354∼2.891)
Bright dormitory light (1:no, 2:yes)	0.950 (0.644∼1.401)^*^	1.429 (1.031∼1.980)
Dormitory environmental hygiene (1:good, 2:general, 3:bad)	1.406 (1.021∼1.937)	1.428 (1.069∼1.907)
Monthly expenses of using mobile phones (1: ≤50 RBM, 2: >50 RBM)	1.833 (1.240∼2.709)	1.126 (0.822∼1.542)^*^
Time spent on mobile phones before sleep (1: ≤45 min, 2: >45 min)	1.624 (1.081∼2.438)	2.135 (1.516∼3.008)
Smoking (1:never, 2: occasional, 3:often)	1.677 (1.219∼2.306)	2.577 (1.034∼6.424)
Drinking (1:never, 2: occasional, 3:often)	1.373 (0.958∼1.969)^*^	1.648 (1.197∼2.269)
Coffee intake before sleep (1:never, 2: occasional, 3:often)	2.109 (1.253∼3.550)	1.989 (1.111∼3559)
Skipping breakfast (1:never, 2:1or 2 times/week, 3: ≥3 times/week)	1.161 (0.880∼1.532)^*^	1.593 (1.256∼2.022)
Night snack intake (1:never, 2:1or 2 times/week, 3: ≥3 times/week)	1.498 (1.099∼2.041)	1.138 (0.859∼1.509)^*^
Physical condition (1:good, 2:general, 3:bad)	2.865 (1.981∼4.145)	2.676 (1.956∼3.661)
Time spent on playing games (1:never, 2:1–2 hours/day, 3:3–4 hours/day, 4: ≥4 hours/day)	1.041 (0.834∼1.300)^*^	1.421 (1.126∼1.793)
Physical exercise (1: ≥5 times/week, 2:3 or 4 times/week, 3:1 or 2 times/week, 4:never)	1.239 (1.005∼1.527)	1.207 (0.998∼1.459)^*^
Relationship with classmates (1:harmony, 2: general, 3:strained)	1.366 (0.934∼1.998)^*^	1.674 (1.182∼2.371)
Academic stress (1:mild, 2: moderate, 3: severe)	1.209 (0.866∼1.687)^*^	1.532 (1.134∼2.071)
Family economic conditions (1:affluence, 2: general, 3:poverty)	1.062 (0.753∼1.499)^*^	1.461 (1.047∼2.039)

**Notes.**

* *P* > 0.05.

**Table 4:** The multivariate logistic regression analysis.

Gender	Variables	*P* value	*OR*	95% *CI*
Male	Constant	<0.001	0.073	
	Smoking	0.001	1.754	1.258∼2.446
	Physical condition	<0.001	2.971	2.034∼4.339
Female	Constant	<0.001	0.003	
	Coffee intake before sleep	0.015	2.111	1.155∼3.861
	Skipping breakfast	0.028	1.332	1.031∼1.721
	Time spent on mobile phones before sleep	0.003	1.745	1.210∼2.515
	Physical condition	<0.001	2.507	1.797∼3.497
	Time spent on playing games	0.020	1.347	1.048∼1.730
	Dormitory noise	0.001	2.025	1.354∼3.030
	Academic stress	0.008	1.561	1.126∼2.166

## Discussion

In our study, participants were between 17 and 26 years old, while the age of most Chinese undergraduates was usually from 18 to 23 years old, but there were only 1% (11/1063) participants aged 24 to 26, and we can not exclude data as they really were undergraduates. The reason for participants over 24 years old might be that quiet a number of students, especially in rural areas, started their primary education late for the lack of tutors, and a small number of students participated *gaokao*, the standardized National College Entrance Examination, repeatedly until they were admitted to a preferred college, which all resulted in that a few participants were admitted more than 18 years old and still pursued a undergraduate degree at age 26.

The results in this survey showed that the prevalence rate of poor sleep quality among college students was 53.7% (571/1063) with an average PSQI score of 5.94 ± 2.73, which is higher than that in one previous research (Li et al., 2018), using same methodology (PSQI >5), 47.2% (8722/19654); the reason might due to the lower income and relative laggard compared to other parts of China (Peltzer & Pengpid, 2015), but the rate was similar with the rate reported in a study (Wang et al., 2019), 52.08% (213/409), using same tools and cut-off scores (PSQI >5) to determine sleep disturbances. Considering the different cut-off score of PSQI in previous studies, in order to ensure comparability, we compared two meta-analysis studies using same tools and similar cut-off score (PSQI >7), the prevalence rate in undergraduates (21.1%) was significantly higher than that in general population (15.1%) (Cao et al., 2017), which suggested that measures should be carried out to improve the sleep quality of college students.

In addition, there were statistically significant differences in sleep quality between sex, such as the general score of PQSI, bedtime, get up time, subjective sleep quality, sleep latency, sleep disturbance, and daytime dysfunction. A previous study reported that men tended to have better subjective sleep quality, longer sleep time and shorter sleep latency (Jin et al., 2014), which was same as the results of our study. Glavin, Matthew & Spaeth (2022) also found that women reported earlier bedtime than men. It has been confirmed that women have predisposition of poor sleep. Irregular menstrual cycle (Nam, Han & Lee, 2017) and menstrual pain (Kazama, Maruyama & Nakamura, 2015) would also negatively affect women’s sleep duration, which would cause poor sleep quality for women.

When the relationship between sleep quality and grades was examined, there was a statistically significant difference. Freshmen had better sleep quality than that of sophomores and juniors, which might attribute to the fact that the survey was conducted during December, since most courses finished around this time, there were more exams than usual; Part of the courses ended earlier and had finished the final exams before December, therefore students just needed review the subjects finished but not tested while attending classes to continue with courses not yet completed. Freshmen entered university in late September, there were only three months in the autumn semester from October to mid-January, to help them better adapt to college life; there were more club activities on campus to attend and fewer courses to learn than sophomores and juniors, so they suffered relatively mild test pressure. While sophomores suffered the worst sleep quality, it might be due to the ease study of freshmen and the intensive learning of sophomores. After a year of adaptation and adjustment, the sleep quality of sophmores had been improved.

Meanwhile, this survey found that the sleep quality of medical students was worse than that of non-medical students, and there were significant differences in sleep latency, daytime dysfunction and bedtime between majors. Nadeem A also reported that medical students tended to have worse sleep than non-medical students (Nadeem et al., 2018). The reasons might contain the vast range of academic courses, the high level of clinical work intensity, the overnight and on-call shifts during the internship for medical students to achieve the necessary professional knowledge and skills (Sun et al., 2022).

Considering the differences of life habits between sex, such as smoking, drinking, the amount and frequency of exercise, personal hygiene and the frequency of playing games, which were all associated with sleep quality, the influence factors between genders were analyzed respectively.

The results of multivariate logistic regression analysis displayed that the influence factors of male sleep quality consisted of smoking and health status, the worse health status they got and the more cigarette they smoked, the worse sleep quality they suffered. Similarly, the study of Nasri et al. (2021) found that both active smoking and exposure to secondhand smoke would have a negative impact on sleep quality. This phenomenon might attribute to the fact that the direct effect of nicotine on sleep-wake cycle neurons can cause sleep disturbance in smokers, showed by prolonged sleep onset latency, higher dopamine levels and lower dopamine transporter levels in cerebrospinal fluid of active smokers (Li et al., 2020).

The research also indicated that the influence factors of female sleep quality covered poor health status, server study pressure, dormitory noise, skipping breakfast, drinking coffee before sleep, longer time spent on mobile phones before sleep, and longer time of playing games per day. This investigation reported that health status was an important influence factor of sleep quality. A survey (Wu et al., 2015) suggested that there was a close relationship between sleep quality and physical activity and it was of great importance to participate physical exercise regularly to improve sleep quality. The frequency and usage time of mobile phones before sleep had a significant impact on the sleep quality of female college students, which was consistent with the research results of Wang et al. (2019). Mobile phones have become an integral part of students’ quotidian lives, a survey reported that one hundred percent of the college students had mobile phones and used mobile phones (Meng et al., 2021), and another the prevalence of problematic mobile phone use has been found to be 28.2% among Chinese college students (Tao et al., 2017). Additionally, long-term and excessive usage of mobile phones could easily lead to musculoskeletal discomfort (Yang et al., 2017) and contribute to higher interpersonal conflicts (Ellahi et al., 2021), which may directly or indirectly affect sleep quality. Furthermore, when they use mobile phones before going to bed, people will expose to blue light. Ostrin, Abbott & Queener (2017) informed that exposure of increasing amounts of artificial light during the night may contribute to the high prevalence of reported sleep dysfunction, and computers and handheld devices could release blue light. The PSQI score of students who held the phone at a distance of more than 10 cm was found significantly higher than those who held it at a distance of less than 10 cm (Meng et al., 2021). In addition, it is essential of a good sleep environment to ensure sleep quality. Because of many students living in the same dormitory, different timetable and living habits, lack of effective supervision, and the phenomenon of playing games or chatting before sleep had caused dormitory noises, all the factors were detrimental to ensure sleep quality. The survey reported that there was a significant association between study pressure and sleep quality. Similarly, a systematic review found close relationships between sleep quality and/or insomnia with stress in students (Gardani et al., 2022). Drinking coffee before going to bed and skipping breakfast would affect sleep quality as well, which was the same as previous research results (Sawah et al., 2015; St-Onge, Mikic & Pietrolungo, 2016). Drinking coffee before going to bed was likely to cause sleep disorders, especially difficulties in falling asleep, because coffee is an excitatory drink, and it is easy of drinking coffee before going to bed to cause excessive excitement in the brain. Skipping breakfast could cause blood sugar instability, and hypoglycemic status could result in lethargy and dysfunction during the day. Moreover, there were more influence factors of sleep quality of females than those of males, which indicated that girls were more easily affected than boys, and needed further attention.

There were three shortcomings in this study. On one hand, all sleep and behavioral data in this study were self-reported, so there might be bias (for example, recall bias); on the other hand, since it was a cross-sectional study, we can only establish associations between variables, and not infer causation. Moreover, in our study, the self-reported and fewer items to evaluate psychological factors related to sleep quality, which may be overestimated or undervalued its intensity. While the investigation suggested several factors that may influence sleep quality among undergraduates, and the results had provided bases for working out measures to improve sleep quality of university students. The measures are listed as follows: First, students should be called on to strengthen physical exercise and cut down energy drink intake to improve health status; second, students need to help each other, formulate plan, and cultivate interests in study, which aims to improve grades and relieve study pressure; third, it is necessary to carry out some measures to strengthen dormitory management to improve dormitory hygiene; furthermore, publicity campaigns can be conducted to explanation the detriment of using mobile phones before going to bed and the importance of regular meals; moreover, they can listen to some soothing music before sleep.

## Conclusions

The investigation reported about half of college students were poor sleep. Women, medical students, and sophomores have a predisposition of poor sleep. Moreover, there were more risk factors associated with the poor sleep quality of women than that of men. Health policymakers should fully consider these factors in improving the sleep quality of university students.

##  Supplemental Information

10.7717/peerj.13833/supp-1 Supplemental Information 1 Raw data

10.7717/peerj.13833/supp-2 Supplemental Information 2 Questionnaire

10.7717/peerj.13833/supp-3 Table S1 The univariate analysis of influencing factors related to sleep quality among males

10.7717/peerj.13833/supp-4 Table S2 The univariate analysis of influencing factors related to sleep quality among females

## Additional Information and Declarations

### Data Availability

The following information was supplied regarding data availability: The raw measurements are available in the Supplementary Files.

## References

[ref-1] Barahona-Correa JE, Aristizabal-Mayor JD, Lasalvia P, Ruiz ÁJ, Hidalgo-Martínez P (2018). Sleep disturbances, academic performance, depressive symptoms and substance use among medical students in Bogota, Colombia. Sleep Science.

[ref-2] Buysse D, Reynolds C, Monk T, Berman S, Kupfer D (1989). The Pittsburgh sleep quality index: a new instrument for psychiatric practice and research. Psychiatry Research.

[ref-3] Cao X, Wang S, Zhong B, Zhang L, Ungvari G, Ng C, Li L, Chiu H, Lok G, Lu J, Jia F, Xiang Y (2017). The prevalence of insomnia in the general population in China: a meta-analysis. PLOS ONE.

[ref-4] Chimed-Ochir O, Ando S, Murakami S, Kubo T, Ishimaru T, Fujino Y, Ikaga T (2021). Perception of feeling cold in the bedroom and sleep quality. Nagoya Journal of Medical Science.

[ref-5] Cohrs S, Rodenbeck A, Riemann D, Szagun B, Jaehne A, Brinkmeyer J, Gründer G, Wienker T, Diaz-Lacava A, Mobascher A, Dahmen N, Thuerauf N, Kornhuber J, Kiefer F, Gallinat J, Wagner M, Kunz D, Grittner U, Winterer G (2014). Impaired sleep quality and sleep duration in smokers—results from the German Multicenter Study on Nicotine dependence. Addiction Biology.

[ref-6] Ellahi A, Javed Y, Begum S, Mushtaq R, Rehman M, Rehman HM (2021). Bedtime smart phone usage and its effects on work-related behaviour at workplace. Frontiers in Psychology.

[ref-7] Fatima Y, Doi SAR, Al Mamun A (2018). Sleep problems in adolescence and overweight/obesity in young adults: is there a causal link?. Sleep Health.

[ref-8] Gardani M, Bradford D, Russell K, Allan S, Beattie L, Ellis J, Akram U (2022). A systematic review and meta-analysis of poor sleep, insomnia symptoms and stress in undergraduate students. Sleep Medicine Reviews.

[ref-9] Glavin E, Matthew J, Spaeth A (2022). Gender differences in the relationship between exercise, sleep, and mood in young adults. Health Education & Behavior: the Official Publication of the Society for Public Health Education.

[ref-10] Jiang XL, Zheng XY, Yang J, Ye CP, Chen YY, Zhang ZG, Xiao ZJ (2015). A systematic review of studies on the prevalence of insomnia in university students. Public Health.

[ref-11] Jin Y, Ding Z, Fei Y, Jin W, Liu H, Chen Z, Zheng S, Wang L, Wang Z, Zhang S, Yu Y (2014). Social relationships play a role in sleep status in Chinese undergraduate students. Psychiatry Research.

[ref-12] Kazama M, Maruyama K, Nakamura K (2015). Prevalence of dysmenorrhea and its correlating lifestyle factors in Japanese female junior high school students. The Tohoku Journal of Experimental Medicine.

[ref-13] Kenney S, Lac A, Labrie J, Hummer J, Pham A (2013). Mental health, sleep quality, drinking motives, and alcohol-related consequences: a path-analytic model. Journal of Studies on Alcohol and Drugs.

[ref-14] Lee J (2017). Sleep duration’s association with diet, physical activity, mental status, and weight among Korean high school students. Asia Pacific Journal of Clinical Nutrition.

[ref-15] Li H, Liu Y, Xing L, Yang X, Xu J, Ren Q, Su KP, Lu Y, Wang F (2020). Association of cigarette smoking with sleep disturbance and neurotransmitters in cerebrospinal fluid. Nature and Science of Sleep.

[ref-16] Li X, Tan Y, Li S, Wang X (2022). Psychological distress and smoking behaviors of Chinese college students: mediating effects of the dimensions of learning burnout. BMC Psychology.

[ref-17] Li L, Wang Y, Wang S, Zhang L, Li L, Xu D, Ng C, Ungvari G, Cui X, Liu Z, De Li S, Jia F, Xiang Y (2018). Prevalence of sleep disturbances in Chinese university students: a comprehensive meta-analysis. Journal of Sleep Research.

[ref-18] Meng J, Wang F, Chen R, Hua H, Yang Q, Yang D, Wang N, Li X, Ma F, Huang L, Zou Z, Li M, Wang T, Luo Y, Li Y, Liu YA-O (2021). Association between the pattern of mobile phone use and sleep quality in Northeast China college students. Sleep and Breathing.

[ref-19] Nadeem A, Cheema M, Naseer M, Javed H (2018). Comparison of quality of sleep between medical and non-medical undergraduate Pakistani students. The Journal of the Pakistan Medical Association.

[ref-20] Nam G, Han K, Lee G (2017). Association between sleep duration and menstrual cycle irregularity in Korean female adolescents. Sleep Medicine.

[ref-21] Nasri O, Pouragha H, Baigi V, Shalyari N, Yunesian M (2021). Quality of life and sleep disorders in Tehran Employees Cohort (TEC); Association with secondhand smoking and wealth index. Journal of Environmental Health Science and Engineering.

[ref-22] Ostrin LA, Abbott KS, Queener HM (2017). Attenuation of short wavelengths alters sleep and the ipRGC pupil response. Ophthalmic and Physiological Optics.

[ref-23] Öztürk ME, YabancıAyhan N (2017). Associations between poor sleep quality, obesity, and the anthropometric measurements of women in Turkey. Ecology of Food and Nutrition.

[ref-24] Peltzer K, Pengpid S (2015). Nocturnal sleep problems among university students from 26 countries. Sleep & Breathing.

[ref-25] Pilz LK, Keller LK, Lenssen D, Roenneberg T (2018). Time to rethink sleep quality: PSQI scores reflect sleep quality on workdays. Sleep.

[ref-26] Raniti MB, Waloszek JM, Schwartz O, Allen NB, Trinder J (2018). Factor structure and psychometric properties of the Pittsburgh Sleep Quality Index in community-based adolescents. Sleep.

[ref-27] Sawah M, Ruffin N, Rimawi M, Concerto C, Aguglia E, Chusid E, Infortuna C, Battaglia F (2015). Perceived stress and coffee and energy drink consumption predict poor sleep quality in podiatric medical students. A cross-sectional study. Journal of the American Podiatric Medical Association.

[ref-28] Shadzi M, Salehi A, Vardanjani H (2020). Problematic internet use, mental health, and sleep quality among medical students: a path-analytic model. Indian Journal of Psychological Medicine.

[ref-29] St-Onge MP, Mikic A, Pietrolungo CE (2016). Effects of diet on sleep quality. Advances in Nutrition.

[ref-30] Sun Y, Wang H, Jin T, Qiu F, Wang X (2022). Prevalence of sleep problems among chinese medical students: a systematic review and meta-analysis. Frontiers in Psychiatry.

[ref-31] Swinkels CM, Ulmer CS, Beckham JC, Buse N, Calhoun PS (2013). The association of sleep duration, mental health, and health risk behaviors among U.S. Afghanistan/Iraq era veterans. Sleep.

[ref-32] Tao S, Wu X, Zhang Y, Zhang S, Tong S, Tao F (2017). Effects of sleep quality on the association between problematic mobile phone use and mental health symptoms in chinese college students. International Journal of Environmental Research and Public Health.

[ref-33] Wang PY, Chen KL, Yang SY, Lin PH (2019). Relationship of sleep quality, smartphone dependence, and health-related behaviors in female junior college students. PLOS ONE.

[ref-34] Wu X, Tao S, Zhang Y, Zhang S, Tao F (2015). Low physical activity and high screen time can increase the risks of mental health problems and poor sleep quality among Chinese college students. PLOS ONE.

[ref-35] Yang SY, Chen MD, Huang YC, Lin CY, Chang JH (2017). Association between smartphone use and musculoskeletal discomfort in adolescent students. Journal of Community Health.

[ref-36] Yang S, Wang Y, Lee Y, Lin Y, Hsieh P, Lin P (2022). Does smartphone addiction, social media addiction, and/or internet game addiction affect adolescents’ interpersonal interactions?. Healthcare.

[ref-37] Yu Y, Lau J, Li J, Mo P, Li J, Chen Y, Ma L (2022). Very tall female young adults tended to be smokers: a large-scale exploratory cross-sectional survey of 26,405 college students in China. Journal of American College Health.

