# Supplementary material for: Deteriorated sleep quality and influencing factors among undergraduates in northern Guizhou, China
Source: PeerJ. 2022 Aug 24;10:e13833. doi: 10.7717/peerj.13833 (PMC9419714; doi:10.7717/peerj.13833)
Supplement: Supplemental Information 2 [file peerj-10-13833-s002.pdf]

附件一：

编号：_______ 调查员____

关于我校大学生睡眠状况及影响睡眠因素的调查问卷

亲爱的同学：

您好，这是一份关于大学生睡眠状况及影响睡眠因素的调查问卷，您的回答对我们有很大的帮助，希望占用您几分钟时间，非常感谢您的配合！您的资料仅用于数据分析，将不会用做其他用途！谢谢合作！

性别：1 男　2 女　年龄：______岁　身高：_____cm　体重：_____Kg　年级：______级

专业：____________　类别：1 二本　2 三本

匹兹堡睡眠质量调查问卷（PSQI 量表）

下面一些问题是关于您最近 1 个月的睡眠情况，请选择或填写最符合您近 1 个月实际情况的答案。请回答下列问题！

1.晚上上床睡觉通常 ____点钟。

2.从上床到入睡通常需要____分钟。

3.通常早上____点起床

4.每夜通常实际睡眠_____小时(不等于卧床时间)。

5.您如有因下列情况影响您的睡眠而烦恼，请在相应格子内打勾：

	**无**	**＜1 次/周**	**1-2 次/周**	**≥ 3 次/周**
入睡困难(30 分钟内不能入睡)				
夜间易醒或早醒				
夜间去厕所				
呼吸不畅				
咳嗽或鼾声高				
感觉冷				
感觉热				
做恶梦				
疼痛不适				
其它影响睡眠的事情				
如有，请说明：				

6.总的来说，您认为自己的睡眠质量　a.很好　b.较好　c.较差　d.很差

7.您用药物催眠的情况　a.无　b.＜1 次/周　c.1-2 次/周　d.≥ 3 次/周

8.您常感到困倦吗　a.无　b.＜1 次/周　c.1-2 次/周　d.≥ 3 次/周

9.您做事情的精力不足吗　a.没有　b.偶尔有　c.有时有　d.经常有

影响睡眠的因素（自制）

1.您是否吸烟　　a.从不　　b.偶尔（每周少于 1 次）　　c.经常

2.您是否饮酒　　a.从不　　b.偶尔（每月少于 1 次）　　c.经常

3.您是否睡前喝咖啡　　a.从不　　b.偶尔（每月少于 2 次）　　c.经常

4.您睡前吃宵夜的情况　　a.从不　　b.每周 1-2 次　　c.每周 3 次以上

5.您不吃早餐的情况　　a.从不　　b.每周 1-2 次　　c.每周 3 次以上

6.您认为自己的健康状况　　a.好　　b.一般　　c.差

7.您使用手机的情况

7.1 平均每天使用手机的时间　　a.1h 以下　　b.1-3h　　c.3-5h 以上　　d.5h 以上

7.2 平均每月使用手机的费用　　a.小于 50 元　　b.大于 50 元

7.3 睡前玩手机多长时间可以入睡？　约__________分钟

8.您平均每天使用电脑的时间　　a.0-2h/天　　b.2-4h/天　　c.4-6h/天　　d.6h 以上/天

9.您每天打游戏的情况为　　a.从不　　b.每天 0-2h　　c.每天 2-4h　　d.每天 4h 以上

10.您参加体育锻炼的频率为　　a.不锻炼　　b.1-2 次/周　　c.3-4 次/周　　d.5 次以上

11. 宿舍环境：

11.1 您认为宿舍舍友睡觉晚吗？　　a.是　　b.否

11.2 您认为宿舍嘈杂吗？　　a.是　　b.否

11.3 您认为宿舍灯光亮吗？　　a.是　　b.否

11.4 您认为宿舍环境卫生怎样？　　a.好　　b.一般　　c.差

12.您认为与同学关系如何？　　a.相处融洽　　b.相处一般　　c.不好相处

13.您觉得学习压力如何？　　a.大　　b.一般　　c.小

14.您决定考研吗？　　a.是　　b.否

15.您觉得就业压力大吗？　　a.大　　b.一般　　c.小

16.您认为家庭经济条件如何？　　a.好　　b.一般　　c.差

17.您需要做兼职或其他途径赚取生活费吗？　　a.是　　b.否

18.您有谈恋爱吗？　　a.从没有　　b.曾经有　　c.现在有(若选此项做 19 题，否则跳至 20 题）

19.您认为您与恋人之间的关系如何？　　a.相处融洽　　b.相处一般　　c.不好相处

20.您认为您目前的状况是否需要心理咨询或心理辅导吗？　　a.是　　b.否

21.您认为哪些因素促进了您的睡眠____

22.您认为哪些因素影响了您的睡眠____

调查日期：______年______月______日
