# Supplementary material for: Deteriorated sleep quality and influencing factors among undergraduates in northern Guizhou, China
Source: PeerJ. 2022 Aug 24;10:e13833. doi: 10.7717/peerj.13833 (PMC9419714; doi:10.7717/peerj.13833)
Supplement: Table S1 [file peerj-10-13833-s003.docx]

**Table 1. The Univariate analysis of influencing factors related to sleep quality among male**

| Variables | Category | Good sleep quality | Poor sleep quality | Statistic value | *P* value |
| --- | --- | --- | --- | --- | --- |
| dormitory noise | no | 148（58.3） | 106（41.7） | $\chi^{2}$=5.413 | 0.020 |
|  | yes | 76（46.6） | 87（53.4） |  |  |
| monthly expenses of using mobile phones | ≤50RBM | 139（60.4） | 91（39.6） | $\chi^{2}$=9.310 | 0.002 |
|  | ＞50RBM | 85（45.5） | 102（54.5） |  |  |
| time spent on mobile phones before sleep | ≤45 min | 158（57.9） | 115（42.1） | $\chi^{2}$=5.499 | 0.019 |
|  | ＞45 min | 66（45.8） | 78（54.2） |  |  |
| smoking | never | 188 | 137 | Z=3.248 | 0.001 |
|  | occasional | 23 | 31 |  |  |
|  | often | 13 | 25 |  |  |
| coffee intake before sleep | never | 204 | 159 | Z=2.689 | 0.007 |
|  | occasional | 19 | 28 |  |  |
|  | often | 1 | 6 |  |  |
| night snack intake | never | 60 | 29 | Z=2.588 | 0.010 |
|  | 1-2 times per week | 126 | 123 |  |  |
|  | ≥3 times per week | 38 | 41 |  |  |
| physical condition | good | 127 | 62 | Z=5.528 | ＜0.001 |
|  | general | 96 | 116 |  |  |
|  | bad | 1 | 15 |  |  |
| physical exercise | ≥5 times per week | 49 | 34 | Z=2.085 | 0.037 |
|  | 3-4 times per week | 55 | 37 |  |  |
|  | 1-2 times per week | 100 | 96 |  |  |
|  | never | 20 | 26 |  |  |
| dormitory environmental hygiene | good | 106 | 72 | Z=2.124 | 0.034 |
|  | general | 105 | 105 |  |  |
|  | bad | 13 | 16 |  |  |
