# Supplementary material for: Deteriorated sleep quality and influencing factors among undergraduates in northern Guizhou, China
Source: PeerJ. 2022 Aug 24;10:e13833. doi: 10.7717/peerj.13833 (PMC9419714; doi:10.7717/peerj.13833)
Supplement: Table S2 [file peerj-10-13833-s004.docx]

**Table 2. The univariate analysis of influencing factors related to sleep quality among female**

| Variables | Category | Good sleep quality | Poor sleep quality | Statistic value | *P* value |
| --- | --- | --- | --- | --- | --- |
| dormitory noise | no | 219（45.5） | 262（54.5） | $\chi^{2}$=12.687 | ＜0.001 |
|  | yes | 49（29.7） | 116（70.3） |  |  |
| bright dormitory light | no | 89（36.2） | 157（63.8） | $\chi^{2}$=4.610 | 0.032 |
|  | yes | 179（44.8） | 221（55.3） |  |  |
| time spent on mobile phones before sleep | ≤45 min | 200（47.7） | 219（52.3） | $\chi^{2}$=19.167 | ＜0.001 |
|  | ＞45 min | 68（30.0） | 159（70.0） |  |  |
| smoking | never | 263 | 360 | *Z*=1.969 | 0.049 |
|  | occasional | 5 | 14 |  |  |
|  | often | 0 | 4 |  |  |
| drinking | never | 186 | 219 | *Z*=3.023 | 0.003 |
|  | occasional | 81 | 154 |  |  |
|  | often | 1 | 5 |  |  |
| coffee intake before sleep | never | 59 | 75 | *Z*=2.517 | 0.012 |
|  | occasional | 182 | 258 |  |  |
|  | often | 27 | 45 |  |  |
| skipping breakfast | never | 102 | 99 | *Z*=3.846 | ＜0.001 |
|  | 1-2 times per week | 135 | 201 |  |  |
|  | ≥3 times per week | 31 | 78 |  |  |
| physical condition | good | 122 | 89 | *Z*=6.251 | ＜0.001 |
|  | general | 143 | 267 |  |  |
|  | bad | 3 | 22 |  |  |
| time spent on playing games | never | 156 | 173 | *Z*=3.193 | 0.001 |
|  | 1-2 hours per day | 95 | 167 |  |  |
|  | 3-4 hours per day | 12 | 28 |  |  |
|  | ≥4 hours per day | 5 | 10 |  |  |
| physical exercise | ≥5 times per week | 33 | 39 | *Z*=2.111 | 0.035 |
|  | 3-4 times per week | 52 | 50 |  |  |
|  | 1-2 times per week | 151 | 234 |  |  |
|  | never | 32 | 55 |  |  |
| dormitory environmental hygiene | good | 144 | 168 | *Z*=2.402 | 0.016 |
|  | general | 119 | 198 |  |  |
|  | bad | 5 | 12 |  |  |
| relationship with classmates | harmony | 208 | 257 | *Z*=2.760 | 0.006 |
|  | general | 60 | 116 |  |  |
|  | strained | 0 | 5 |  |  |
| academic stress | mild | 9 | 5 | *Z*=2.698 | 0.007 |
|  | moderate | 168 | 208 |  |  |
|  | severe | 91 | 165 |  |  |
| family economic conditions | affluence | 17 | 11 | *Z*=2.160 | 0.031 |
|  | general | 201 | 277 |  |  |
|  | poverty | 50 | 90 |  |  |
